# The Tendency of Certain Diseases to Die out

**Published:** 1907-11-16

**Authors:** 


### Public Health and Hygiene.

### THE TENDENCY OF CERTAIN DISEASES TO DIE OUT.

In his presidential address to the Epidemiological Section of the Royal Society of Medicine, Dr. Newsholme discusses a phenomenon of great interest in the course of an inquiry into the relations of "Poverty and Disease as Illustrated by the Course of Typhus Fever and Phthisis in Ireland."

In the opinion of Dr. Newsholme, to speak of a "tendency towards extinction" is to propound a "mystical doctrine" of disease which has the appearance of leaving us the victims of blind chance, unless tendency is defined as "applying to factors of whose method of operation we know nothing."

We are so far in agreement with Dr. Newsholme as to believe that "tendency" is a word not to be uttered by the scientific man if he desires to be as accurate in description as he endeavours to be in observation. It is an animistic term; it implies a purpose in the process; it is, applied to a disease of diminishing incidence, as though extinction were an end in view, somehow in the process itself. At the best it is a bad descriptive term, merely asserting of an observed series of events that if continued a certain result can be foretold.

But it makes confusion worse confounded to apply it to factors of whose methods of operation we know nothing. Factors are the same trouble over again—animism in a worse form, and "factors of whose method of operation we know nothing" are even less than what we know of their unthinkable deeds. Dr. Newsholme has not improved the mystical phrase by his attempt at definition. And, indeed, Dr. Newsholme is not at his best in the use and exposition of scientific terminology. A tendency "without some agency to produce it is inconceivable," he tells us. What are agents which produce tendencies? we ask. Surely it is as unscientific to speak of agency in the chain of cause and sequence as it is to speak of tendencies in the same process. But Dr. Newsholme has been less concerned at falling himself into scholastic error than at tilting against those who group an unanalysed mass of conditions and ascribe to it a casual relationship in the series of phenomena they are describing. "No one now," he says, "acquainted as we are with the relation of rats to plague and of mosquitoes to malaria, would hesitate in giving first place to influences inimical to these carriers of infection, in bringing about the disappearance of these diseases from England; though he might be uncertain as to the exact mode by which the link in the chain of infection had been broken in each instance. Similarly, although there may still be differences of opinion as to the relative share borne by increased domestic cleanliness and the extensive segregation of the sick, in producing the extinction of leprosy from this country, we have no doubt that these, and not a vague 'tendency to extinction,' have produced the result."

This at least is plausible; and, indeed, we share with Dr. Newsholme the desire for definite and precise description rather than vague suggestiveness in accounts of natural phenomena. Nevertheless, a statement of ignorance as to the "how comes" of certain obvious facts is often to be preferred to grotesque guesses which narrow the outlook and distract from the broad facts in a pseudo-illumination of obscure detail. Dr. Charles Creighton has suggested that "the substitution of coffin burial for burial in shrouds or cere cloths was responsible for the disappearance of plague, acting, he suggests, by preventing contamination of the soil in the crowded churchyards."

Dr. Newsholme improves on this theory. "I suggest," he says, "the tentative view that the coffin-burial was the more efficient agent in diminishing plague; not, as has been suggested, by diminishing the contamination of urban soil, but by preventing the predation of rats on the buried and uncoffined corpses and their subsequent raids on human food and contamination more generally of dwelling houses." We confess we prefer the more general confession of ignorance in regard to the disappearance of plague from this country which is summed up in the description "tendency towards extinction" to the more precise ascription of its decline to the change from shroud to coffin burial, and the alternative views of Dr. Creighton and Dr. Newsholme as to the mode of operation of this change. Without discussing the intricate problem of the epidemiology of plague we simply do not believe that the change from cere cloth to coffin encasement of the dead was responsible in any appreciable degree for the disappearance of plague from England, and we believe it to be unimaginable in any concrete view of plague, notwithstanding the ingenious speculations of these two distinguished epidemiologists.

Among the diseases showing "a tendency to extinction," the two which chiefly claimed the attention of Dr. Newsholme were typhus and phthisis. With his indefatigable energy and industry he has collected a vast amount of fact and figures which have been ingeniously brought to bear in the elucidation of the problem to which he has set himself. This problem may be described as the influence of "immobilising" infection in diminishing the incidence of these diseases. Dr. Newsholme has for some time propounded the view that the main "factor" in the reduced and diminishing incidence of phthisis is the immobilisation of the infection by the increased and increasing institutional treatment of this disease. He has found confirmation of this view in a study of the "tendency" of these diseases chiefly in Ireland.

Typhus fever has practically been extinguished in this country, and is "tending" toward extinction in Ireland. Phthisis is diminishing in Great Britain and elsewhere, but is increasing in Ireland. How are these facts to be correlated? The variation in the anomalies of Poor-law administration in Ireland, coupled with the relationship of famine to the spread of typhus, give the key to the problem. Famine in Ireland has meant increase of vagrancy and vagrancy has suggested itself "as the most effective of the influences favouring the spread of typhus, and the immobilisation of infection by the diminution of vagrancy and the provision of hospital accommodation as the main cause of diminution of its spread in Ireland. . . The objections of the Irish to the infirmary were easily overcome for this disease (typhus), only exceptionally in the case of phthisis. Hence the same measures which were successful for typhus led to an actual increase of phthisis. Typhus has been brought towards the point of extinction by its institutional treatment, acting in conjunction with the removal of the motives for vagrancy. Phthisis has been rendered even more prevalent than formerly, by increasing for this disease domestic at the expense of institutional treatment, and by thus continuing the enormous number of domestic foci of this disease which are implied by the home-treatment of phthisis among the poor."

These are startling speculations. According to this view the great epidemiologists who had to deal with typhus fever as an actual rather than historical fact missed the main feature of its mode of spread, and directed their attention to the less "efficient agents" in its causation. Notwithstanding the careful studies of Dr. Newsholme, we feel nevertheless constrained to agree with the older views regarding typhus fever—namely, that it was conditioned by overcrowding, dirt, and famine, and that the question of the importation or *de novo* origin of the infection under these conditions was a question of secondary importance. Attempts at immobilising the infection have, it seems to us, proved less successful in extinguishing the disease than the adoption of those other measures which were pointed out as best calculated to effect this result. With the abolition of our worst slum areas, and improvement in the general sanitary condition of our gaols, who can doubt that more has been accomplished than would have been possible by the most rigid restraints which could have been imposed upon the diffusion of the infection by isolation of the infected? It is an inversion of factorial importance—still to speak in the phraseology of Dr. Newsholme—to place immobilisation first, and what is connoted by sanitation second, in their influence upon the extinction of typhus fever. Equally impossible of belief is it that the continuous and phenomenally rapid increase in the migratory habits of our population which have accompanied the extraordinary developments in facilities of travel during the last half-century have in this country been associated with the "immobilisation of infection" in the case of tubercle. The growth of cities, the concentration of population in urban areas, the great development of tenement dwellings, the dense aggregation of people in streets, in buildings, in railway trains, in "trams" and "tubes," with undreamed of increase of mobility of the sufferers themselves, are incompatible with a view of diminished mobility of infection due to the almost negligible effect of the growth of institutional treatment in the last stages of the disease.

It is still true that most of us living under hygienic conditions are free from tubercular lesions because of the protection afforded by our phagocytes. It is still true that those with marked hereditary or acquired dispositions to tubercular infection are lacking in the most essential protection from the disease. It is also true that natural resistance can be strengthened by the improved nutrition and hygienic life which is increasingly possible to the masses of our countrymen, but it is extremely doubtful if the tubercle bacillus is less than ever ubiquitous, or, if it were, whether any appreciable retardation of the extinction of the disease could be ascribed to this fact.

The plain man will want a lot of convincing that on the average he runs a less risk of exposure to tubercular infection to-day than his forefathers did half a century ago. He will even prefer to accept a "tendency to extinction" the outcome of "unknown factors," than to pin his faith to "immobilisation of infection" as the main cause of the disappearance of diseases which raged most furiously when the world went slower and the mechanical difficulties of migration restrained and localised the social intercourse on which, it is presupposed, the diffusion of infection chiefly depended.

